# *“It wasn’t here, and now it is. It’s everywhere"*: fentanyl’s rising presence in Oregon’s drug supply

**DOI:** 10.1186/s12954-022-00659-9

**Published:** 2022-07-11

**Authors:** Sarah S. Shin, Kate LaForge, Erin Stack, Justine Pope, Gillian Leichtling, Jessica E. Larsen, Judith M. Leahy, Andrew Seaman, Daniel Hoover, Laura Chisholm, Christopher Blazes, Robin Baker, Mikaela Byers, Katie Branson, P. Todd Korthuis

**Affiliations:** 1 Comagine Health, 650 NE Holladay Street #1700, Portland, OR 97232 USA; 2 Department of Medicine, Section of Addiction Medicine, Oregon Health and Science University, Portland, OR USA; 3 Acute and Communicable Disease Prevention, Public Health Division, Oregon Health Authority, Portland, OR USA; 4 Injury and Violence Prevention Program, Public Health Division, Oregon Health Authority, Portland, OR USA; 5 School of Public Health, Oregon Health and Science University-Portland State University, Portland, OR USA; 6 Old Town Clinic/Central City Concern, Portland, OR USA; 7 Better Life Partners, Hanover, NH USA; 8 HIV Alliance, Eugene, OR USA

**Keywords:** Fentanyl, Drug supply, Overdose, EMS, Law enforcement, Qualitative, Opioids, People who use drugs, Substance use

## Abstract

**Background:**

Illicit fentanyl has contributed to a drastic increase in overdose drug deaths. While fentanyl has subsumed the drug supply in the Northeastern and Midwestern USA, it has more recently reached the Western USA. For this study, we explored perspectives of people who use drugs (PWUD) on the changing drug supply in Oregon, experiences of and response to fentanyl-involved overdose, and recommendations from PWUD to reduce overdose risk within the context of illicit fentanyl’s dramatic increase in the recreational drug supply over the past decade.

**Methods:**

We conducted in-depth interviews by phone with 34 PWUD in Oregon from May to June of 2021. We used thematic analysis to analyze transcripts and construct themes.

**Results:**

PWUD knew about fentanyl, expressed concern about fentanyl pills, and were aware of other illicit drugs containing fentanyl. Participants were aware of the increased risk of an overdose but remained reluctant to engage with professional first responders due to fear of arrest. Participants had recommendations for reducing fentanyl overdose risk, including increasing access to information, harm reduction supplies (e.g., naloxone, fentanyl test strips), and medications for opioid use disorder; establishing drug checking services and overdose prevention sites; legalizing and regulating the drug supply; and reducing stigma enacted by healthcare providers.

**Conclusion:**

PWUD in Oregon are aware of the rise of fentanyl and fentanyl pills and desire access to tools to reduce harm from fentanyl. As states in the Western USA face an inflection point of fentanyl in the drug supply, public health staff, behavioral health providers, and first responders can take action identified by the needs of PWUD.

**Supplementary Information:**

The online version contains supplementary material available at 10.1186/s12954-022-00659-9.

## Background

Drug overdose deaths from May 2020 to April 2021 exceeded 100,000; the highest number of overdose deaths recorded over 12 months in the USA [1]. Sixty-four percent of these deaths are estimated to have involved synthetic opioids other than methadone [1]. Non-pharmaceutical fentanyl analogs are an illicitly manufactured class of synthetic opioids that are largely responsible for this increase in fatal overdoses [2]. Overdose deaths involving illicit fentanyl rose 94% in the Western USA between July 2019 and December 2020, the highest rate of any geographic region [1]. Local surveillance data from Oregon and Idaho confirm that fentanyl has entered the regional drug supply [3, 4]. In the Western USA, fentanyl is increasingly pressed into counterfeit pills that resemble and are often sold as oxycodone, alprazolam (Xanax), or other prescription drugs [5, 6].

PWUD in regions with earlier fentanyl saturation are aware of the changing drug supply [7] and the dangers of fentanyl and have made suggestions for reducing harm and risk of overdose. These suggestions include non-law enforcement emergency response to overdose [8]; titration of naloxone in a medical setting to ease withdrawal [9]; respect and empathy within the healthcare system [9, 10]; access to syringe service programs and harm reduction tools [11, 12]; expansion of treatment centers, detox facilities, and housing shelters [10]; and peer-delivered services and information [11]. Studies also show high levels of interest in access to overdose prevention sites [13, 14].

While PWUD welcome tools and information to prevent and reverse an overdose, mistrust and previous negative experiences with healthcare and law enforcement may decrease willingness among PWUD to call 911 during or after an overdose [9, 12, 15]. PWUD report high levels of enacted and anticipated stigma among healthcare providers [12]. Good Samaritan laws in 41 US states grant legal protection to PWUD who call 911 during an overdose; however, the laws offer limited immunity, and many PWUD are either unaware or untrusting of Good Samaritan laws [15]. A recent study found that 39% of PWUD were concerned about an arrest for calling or being at the scene of an overdose and 23% felt vulnerable to arrest if they overdosed; perceived vulnerability to arrest was higher among people of color (51%) [16]. Whether risk–benefit perspectives about calling 911 have shifted in the context of fentanyl’s emerging presence in Oregon is yet unknown.

Oregon is leading the nation with percent of population reporting illicit drug use (2^nd^ in the USA) and methamphetamine use (1st in the USA) [17]. Oregon saw a 306% increase in fentanyl-related overdose deaths from 2019 to 2020 [4]. While exposures to heroin containing fentanyl have been documented throughout the nation [5, 18–21], less is known about how PWUD are impacted by other illicit drugs and counterfeit pills that contain fentanyl in the Western USA, especially in communities with high rates of methamphetamine use and the co-use of methamphetamine and heroin. The degree to which fentanyl is deliberately adulterated (i.e., intentionally added to enhance or mimic the effects of another substance) or accidentally cross-contaminated (i.e., unintentionally added during the handling process) is yet unknown [22]. However, with increases in drug overdose deaths involving fentanyl [1] and drug seizures containing fentanyl [6] it is clear that fentanyl is increasingly present in the illicit drug supply. Fentanyl’s rapid incursion in the Oregon drug supply increases the need to bring awareness to fentanyl, including counterfeit pills containing fentanyl, the symptoms of fentanyl-involved overdose, techniques for responding to overdose in a timely and effective manner, and system-level changes needed to reduce the risk of overdose and overdose death. This study aims to explore PWUD’s understanding of the changing Oregon drug supply, experiences with and response to overdose, and recommendations for communities from PWUD.

## Methods

We conducted rapid assessment semi-structured qualitative interviews with PWUD from seven Oregon counties (Clatsop, Deschutes, Josephine, Lane, Marion, Multnomah, and Umatilla). We selected counties with high rates of overdose and High-Intensity Drug Trafficking Area (HIDTA) drug seizures involving fentanyl, counties with strong community connections to support recruitment, and counties geographically dispersed across urban and rural Oregon. We developed the interview guide through iterative discussions with the research team and community organization staff, including people with lived experience of drug use. We conducted phone interviews to explore knowledge of fentanyl in the drug supply, experiences of unintentional exposure to fentanyl and suspected fentanyl-involved overdose, and suggestions by PWUD to reduce harms from fentanyl. The study was approved by the OHSU Institutional Review Board and granted a Federal Certificate of Confidentiality.

### Participants and procedures

We recruited participants (*N* = 34) from May 11 to June 25, 2021. We recruited three to six participants from each of the five rural and two urban counties. To recruit, we partnered with syringe service programs and local programs that provide harm reduction and recovery peer support. Program staff distributed flyers and recruited via word of mouth, providing potential participants with the research staff phone number to complete the screening questions and interview. Participants received a $50 gift card for participation. Eligibility included: (1) use of methamphetamine, cocaine, benzodiazepines, heroin, illicit fentanyl, or other opioids in the past 30 days and (2) age 18 or greater. Research staff screened participants via phone and, if participants were eligible, started the interview immediately following the screening questions. Interviews lasted about 60 min. Local syringe service programs and peer support programs provided access to telephones for potential participants lacking the means to participate. Research staff obtained verbal consent before data collection.

Four research staff (SS, KL, JP, JEL) conducted all eligibility screenings and interviews. All staff had previous experience and training in qualitative data collection. Three staff had experience interviewing PWUD. Interviewers were trained to follow a protocol if a participant expressed thoughts of suicide, including providing the Suicide Lifeline number, offering to connect the participant to an on-call clinician for crisis counseling, and offering to connect the participant to a peer recovery support specialist for support. Study leadership reviewed audio interview recordings regularly to provide feedback and ensure interview quality and completeness. The interview team met weekly during data collection to discuss interview content. Audio-recorded interviews were transcribed by a professional transcriptionist and uploaded into NVivo software (Version 12) for analysis.

### Analysis

We used thematic analysis with a deductive coding structure to analyze the interviews [23, 24]. We used the interview guide to create the initial codes and an iterative process to refine the codebook and achieve acceptable interrater reliability. Two team members (KL, SS) coded the same transcript and ran a coding comparison query. The first test yielded a low kappa coefficient, so coders (KL, SS) reviewed discrepant codes and added clarity to codebook definitions. Coders then coded a second transcript and ran a coding comparison query, achieving a kappa coefficient of > 0.80, which was deemed sufficient. Coders added additional clarity to codebook definitions and coded the remaining transcripts independently, coding simultaneously and checking in regularly to discuss processes and resolve any coding discrepancies. Coded data were then used to construct themes through an iterative inductive process by four team members (KL, SS, JP, ES). Within themes, subthemes were identified, and relationships between and across themes were examined, such as experiences of fentanyl-involved overdose and engagement with emergency services. These themes were further refined during investigator team discussions.

## Results

Of the 34 participants, most identified as female (47%) or male (47%), age ≥ 30 years (91%), and non-Hispanic White (74%). In the past month, 32 (94%) participants reported non-pharmaceutical methamphetamine or other non-medical stimulant use, 28 (82%) participants reported heroin use, 21 (62%) participants reported non-pharmaceutical fentanyl use, 16 (47%) participants reported non-medical benzodiazepine use, and 14 (44%) participants reported non-medical use of prescription opioids. Twenty-eight (82%) participants reported using heroin and methamphetamine in the past month. Thirty (88.2%) participants reported injection drug use in the past 30 days (Table 1). We constructed three themes from this data: (1) participant reports of changes in the Oregon drug supply and demographics who use fentanyl, (2) participant experiences with and impressions of professional first responders influenced overdose response behavior, and (3) participant recommendations for state and communities (Additional file 1: Table S2).

**Table 1:** Participant demographics and past 30-day drug use (*n* = 34)

Characteristic	Count (%)
Gender	
Female	16 (47%)
Male	16 (47%)
Non-binary	2 (6%)
Age	
< 30	3 (9%)
30–39	15 (44%)
40–49	11 (32%)
50 +	5 (15%)
Race	
White	29 (85%)
Multiracial	3 (9%)
African American or Black	1 (3%)
Other	1 (3%)
Ethnicity	
Non-Hispanic	28 (82%)
Hispanic	6 (18%)
Past 30-day drug use	
Methamphetamine/stimulants	31 (91%)
Heroin	28 (82%)
Street fentanyl*	21 (62%)
Benzodiazepines	16 (47%)
Prescription opioids	15 (44%)
Cocaine or crack*	8 (24%)
Polysubstance use	
Any opioid and any stimulant	28 (82%)
Injection drug use in past 30 days	
Yes	30 (88%)
No	4 (12%)

*Missing 1 response

### Participant reports of changes in the Oregon drug supply and demographics who use fentanyl

Participants described an increased presence of fentanyl in the Oregon drug supply, expansion of drug types that contain fentanyl, and a changing demographic of people using fentanyl. Participants became aware of the shifting drug supply from personal experiences (e.g., unintentional exposure to fentanyl) and conversations with others (e.g., peers and staff at harm reduction agencies).

Participants reported fentanyl entering the drug supply in two main ways: (1) being mixed into drugs such as heroin or methamphetamine and (2) counterfeit pressed pills containing fentanyl. While known identification of fentanyl varied (e.g., dealer disclosure, identification through urinalysis testing, fentanyl test strips, self-identification), participants overwhelmingly reported that the presence of fentanyl is increasingly pervasive in Oregon’s drug supply. One participant, who had been using heroin for over 27 years, described, *“It [fentanyl] wasn’t here, and now it is. It’s everywhere.”* Most believed that there was a strong chance that fentanyl was in the drugs they purchased.

Participants reported that counterfeit pills containing fentanyl were widely available and marketed by dealers as prescription drugs like Oxycodone, Vicodin, or Xanax. One of the commonly available fentanyl pressed pills was described to be small, round, and blue, colloquially referred to as “fetties,” “30 blues,” or “dirty 30 s.” As participants described:

“*It’s [drug supply] been flooded. There’s been a lot of fentanyl, a lot. There are mainly the OxyContin 30-milligram pills, the little, round, blue ones. There’s been a crap-ton of those just flooding the market—a ton of them. Pretty much, if you get an Oxy 30, you know it’s fentanyl. There are no real ones, hardly, going around. It’s just the fentanyl ones.*

*“I know they’re pressing them [fentanyl] in pills a lot, trying to pass them for Vicodin and prescription Oxys [Oxycontin].*”

*“I guess the big one is it seems the Xanax is replaced with it [fentanyl]. It’s causing more and more overdoses because there’s fentanyl in it.*”

Most participants had heard of or had personal experience with heroin, methamphetamine, and other illicit drugs containing fentanyl. One participant, who co-used methamphetamine and heroin, described purchasing heroin in the current market as unpredictable: “*you never know what you’re going to get,”* even when buying from the same source. Other participants shared:

“*Three years ago, four years ago, I would have never known to ask if fentanyl was in the heroin I was buying... Today, there’s more fentanyl-heroin than there is just regular heroin. It’s harder to find regular heroin than it is to find fentanyl. Fentanyl has flooded the market.*”

“I* have heard a lot of people who use meth, saying that they think that there’s fentanyl in it [methamphetamine].*”

Participants observed shifting demographics related to fentanyl incursion in the supply, reporting that people who did not previously use opioids, like young adults and people who use methamphetamine, were using fentanyl or drugs that contain fentanyl. One participant, who used primarily fentanyl, described:

“*A lot of people who were never into opiates, never had a problem with opiates—were just 100 percent meth addicts—they don’t really like it [methamphetamine] now. They don’t hardly ever do meth. It’s all about the fetties [fentanyl pills] and more people—young people, too.*”

Participants also expressed concern about the popularity and desirability of fentanyl among young adults:

“*Been hearing about and seeing [a lot of young people looking for fentanyl] Yeah. Below the age of 21 and 18.*”

“*Well, the younger generation probably is in trouble, because it’s [fentanyl] just starting to get hot and get popular. The younger generation is so influenced by coolness or the popularity-ness or whatever.*”

Participants were aware of a rapid influx of fentanyl in the Oregon drug supply. Participants observed the increasing probability that heroin, methamphetamine, and other illicit drugs may contain fentanyl. Participants expressed concern that the introduction of fentanyl pills imitating pharmaceutical prescription drugs such as opioids or benzodiazepines appealed to young people and people who use methamphetamine.

### Participant experiences with and impressions of professional first responders influenced overdose response behavior

Participants reported experiencing, witnessing, or hearing about suspected fentanyl-involved overdoses. Participants described continued hesitancy to call 911 during an overdose and used alternative strategies to protect themselves and others.

Participants were concerned about the increased risk of overdose from fentanyl. Most participants had experienced, witnessed, or heard of a suspected fentanyl-involved overdose. One participant describing increases in overdose events said, *“I think everybody who I know who’s a drug user has OD’ed [overdosed] at least once this year.”* Participants who used primarily heroin also expressed that the arrival of fentanyl has increased the frequency of overdosing repeatedly:

“*I have, six different times, overdosed… I haven’t this entire time, and I’ve been an addict for 15 years.*”

“*I overdosed on it [fentanyl] three times that week.*”

When asked about calling 911 for emergency services following a suspected fentanyl-involved overdose, some participants were hesitant to call due to fear of arrest from an existing warrant or violating their parole or probation if law enforcement were to arrive on the scene. As one participant described:

“*Most people who are using have a warrant for their arrest from parole and probation. The last thing anybody’s going to do is call the cops if they don’t have to, so nobody was called.*”

Participants also did not feel the need to contact emergency services because they felt they were able to reverse an overdose. Participants responded to an overdose by administering naloxone or engaging in practices like splashing cold water or physical agitation. As one participant described:

“*I threw water on him, slapped him around a couple times, and he finally came around. I think I had an energy drink, a Red Bull, and I just made him drink it. That seemed to bring him back a little bit. Yeah. I was scared.*”

Participants shared adaptive response strategies to preclude law enforcement from arriving on the scene, such as calling the fire department directly and not mentioning “overdose” during the 911 call. As participants shared:

“*Yes. I called the fire department. That’s usually who I call. I don’t ever call the police. I find the fire department in my county, and I call that number directly.*”

“*I don’t say “OD [overdose].” I’m saying, “Someone’s having a hard time breathing. Someone’s having complications.” Because if you say “OD,” then they have to notify the police because the police are there to “protect,” quote unquote, the fire department/EMTs.”*

Others reported considering calling 911 or going to the hospital only when overdose response practices were unsuccessful, including instances in which multiple doses of naloxone were administered but did not reverse the overdose or naloxone was unavailable. As participants reported:

“*In extreme cases, three [naloxone]. By then, the ambulance is there or whatever.*”

“*She finally started breathing on her own again and stuff, but it took hours after that of her to stop breathing again and having to rouse her and everything to get her to breathe again. It was so bad that I told my son, ‘If we can’t get this in the next minute or two, we need to go to the hospital’” … We didn’t have any Narcan.*”

Many participants were hesitant to call 911 for emergency services during a suspected fentanyl-involved overdose. Individual confidence to attend to an overdose and fear of arrest due to outstanding warrants or parole or probationary status were factors that informed hesitancy to contact emergency services. While some participants strategized ways to adapt overdose response practices to preclude a law enforcement dispatch by summoning help from fire personnel exclusively, others would only consider contacting professional first responders if overdose response practices were unsuccessful or if naloxone was not available.

### Participants made recommendations for the state and communities

Participants shared recommendations for changes to services, policies, and practices to reduce harm from fentanyl. Participants suggested supplying more accessible information about fentanyl, increasing access to harm reduction services and supplies, increasing access to substance use disorder treatment services, including medications for opioid use disorder (MOUD); legalizing drug use, or regulating the supply; and addressing stigma.

Participants were interested in learning more about fentanyl, fentanyl’s presence in the local drug supply, and harm reduction practices when using fentanyl. They shared that information should come in multiple formats, including pamphlets or classes, and should be frequently updated, easy to access, and tailored for various populations, including people with less experience using drugs. As participants explained:

“*Knowledge is power. We need drop-in centers and stuff like that, that have pamphlets and information to show new users how to be safe.*”

“*Right now, with fentanyl becoming an epidemic, I think that any and all classes, information, harm reduction, protocols, anything like that would be available to the public, would probably help a lot.*”

Participants also expressed a desire for information about fentanyl to come from a variety of trusted sources, including harm reduction agencies, parole offices, treatment agencies, medical practices, and recovery meetings:

“*It’s really important, I feel, we need to have more information—more insights from doctors’ offices, from meetings, and other places—about fentanyl, and about how it gets mixed with the heroin, it’s really deadly, and all that.*”

Participants called for increased access to and availability of harm reduction supplies such as naloxone and fentanyl test strips to reduce harm from fentanyl. One participant said, “*They should be more liberal handing out the Narcan and test kits for fentanyl.”* Participants also advocated for on-site drug checking options and establishing overdose prevention sites. As one participant described:

“*Little stations where people can go to a safe location to find out if there’s any [fentanyl] in a product. I think that would be cool. Just have private, little spots that have test strips or whatever. People can try them out without the fear of getting in trouble.*”

Several participants discussed the need for more accessible substance use disorder treatment services, including easier access to MOUD:

“*I think that if it [MOUD] could be over-the-counter, it would save so much people. So many people are constantly looking for Subutex, but don’t have Medicare, Medicaid, or OHP [Oregon Health Plan], or a doctor, or something. It could be more readily available or just over-the-counter, even. You would save so much.*”

Some participants recommended increased awareness of the availability of resources, services, and policies such as the Good Samaritan Law. As one participant described:

“*I think the resources that are out there are pretty good in themselves. I think, maybe, more awareness for users that those resources are there.*

Several participants suggested legalizing drug use would allow for regulation of the drug supply to be more aware of what was in the drugs they were using. For example: *“The only other thing I could possibly think of is complete legalization, and then we’ll actually know what we’re getting.”*

Some participants shared that they were hesitant to engage with medical providers for general medical care due to fear of mistreatment and previous negative experiences. Participants expressed that reducing enacted stigma and mistreatment of PWUD in healthcare settings would increase their likelihood of accessing care. One participant recommended educating medical providers about stigma to encourage PWUD to seek out care from hospitals:

“*…maybe talking to medical professionals, because that’s one of the biggest things. People are afraid to go to the hospital. That’s bullshit. I mean, I’m afraid to go to the hospital because I know how I’m going to get treated.*”

Participants shared ideas to reduce harm from fentanyl, including providing more accessible information about fentanyl, increasing access to harm reduction services and supplies (e.g., naloxone, fentanyl test strips, drug checking services, overdose prevention sites), increasing access to substance use disorder treatment like MOUD, regulating the drug supply through legalization or providing a safe supply, and reducing stigmatizing treatment by medical providers to increase willingness to seek emergency care for an overdose.

## Discussion

Participants described how PWUD in Oregon are currently impacted by the influx of fentanyl in the local drug supply. Participants reported that fentanyl pills and heroin, methamphetamine, and other drugs containing fentanyl are widely available and difficult to avoid. Participants expressed awareness of the increasing presence of fentanyl in the drug supply. Still, they were hesitant to contact 911 during a suspected fentanyl-involved overdose due to fear of a law enforcement dispatch. Participants expressed support for disseminating more information about fentanyl, increasing availability and awareness of and improving accessibility of services (e.g., harm reduction and MOUD) and supplies (e.g., naloxone, fentanyl test strips), establishing drug checking services and overdose prevention sites, legalizing and regulating the drug supply, and reducing stigma enacted by medical providers would decrease harms related to increases in fentanyl in the drug supply.

Participants’ experiences signal that the Oregon drug market is following a similar pattern of fentanyl incursion observed in other US regions (East, Midwest, and South) at a three to four-year delay [18, 25]. These early warning signs provide an opportunity to implement strategies to reduce harm from fentanyl based on lessons learned from other states. As PWUD are navigating this volatile market, easily accessible avenues (e.g., point-of-care, mobile spaces, and direct to consumer) of drug checking devices (e.g., Ramon spectrometer, Fourier-transform infrared spectrometer device, fentanyl test strips) should be made available to identify fentanyl presence for consumers [26]. Information gathered from drug checking services may inform harm reduction behaviors, collect surveillance data, and improve drug supply knowledge, including emerging fentanyl analogs [27–29].

Given the rapid onset of fentanyl, PWUD and other bystanders play a crucial role in reversing a fentanyl-involved overdose. However, with hesitancy to seek emergency medical care and the emergence of fentanyl pills that, if orally ingested, may cause delayed toxicity and require prolonged naloxone infusion (26–39 h) [30], an adaptive overdose response may be required. PWUD are interested and want accessible services and tools to respond to the rapidly changing drug supply. These findings highlight an urgent need to implement PWUD-centered strategies in Western US states to improve willingness to call for an emergency medical response following an overdose, including expanding harm reduction tools distribution [31]; involving peer education in overdose response [8]; and reducing enacted stigma, prejudice, and discrimination related to drug use experienced by PWUD from medical providers [12]. The escalating toxicity of the drug supply has heightened the need for overdose prevention sites that have been shown to reduce overdose deaths and facilitate access to healthcare and social services [32–34].

Consistent with literature, participants described a fear of calling 911 in the event of an overdose that was motivated by concerns about an arrest for existing warrants or parole or probation violations [15, 35, 36]. Oregon’s Good Samaritan Law protects people from arrest due to probation/parole violations or outstanding warrants for drug possession when calling 911 during an overdose event [37]. This study was conducted after the decriminalization of low-level drug possession in Oregon via Measure 110, passed in November 2020 [38]. However, decriminalization and Oregon’s Good Samaritan Law do not protect PWUD at the scene of an overdose from potential criminal sanctions related to drug-induced homicide or charges or warrants unrelated to drugs [39]. Given that the rapid onset and atypical fentanyl overdose symptoms (i.e., wooden chest syndrome) may increase the need for follow-up medical attention and additional doses of naloxone [40–42], strategies to encourage PWUD to call 911. In addition to hesitancy related to possible criminal penalties for being at the scene of an overdose, PWUD in this study also reported reluctance to engage with healthcare providers for general medical care due to fear of experiencing mistreatment and stigma [12, 43]. To rebuild and maintain trust between these communities, addressing burnout, trauma, and fatigue among professional first responders and healthcare providers due, in part, to increased exposure to fentanyl-related overdose events is imperative [9, 44]. Care and intervention following a non-fatal fentanyl overdose can establish an avenue for continued care, and connection to post-overdose services, such as initiation on MOUD, connection to peer recovery support specialists, and access to medical and social care [45, 46]. These results add to the existing literature by highlighting that hesitancy to engage with law enforcement and healthcare providers remains a factor that influences overdose response practices, despite increasing exposure to fentanyl-related overdoses and increasing fentanyl prevalence in the Western US drug supply. These findings highlight an urgent need to develop or expand non-law enforcement emergency response such as behavioral health teams and the upcoming 988 hotline [8, 47]. In addition, reducing the stigma experienced by PWUD and ensuring nonjudgmental care within the healthcare system are necessary to improve willingness to access emergency medical services following an overdose.

The emergence of fentanyl pills and heroin, methamphetamine, and other illicit drugs containing fentanyl in the Western US impacts people who did not previously use opioids, including young adults and people who use methamphetamine. Young adults and people who use methamphetamine may be at an increased risk of fatal overdose from fentanyl due to less opioid tolerance [42, 48], less experience administering naloxone [49], less experience and knowledge of harm reduction practices [50], perceptions of immunity to fentanyl [51], and perceptions that methamphetamine use can prevent or reverse an opioid-related overdose [52]. People new to drug use may not be familiar with or see the need to access harm reduction programs for safer use supplies and overdose prevention education. Washington State has documented a striking increase in fentanyl overdose deaths (driven by fentanyl pills) among youth and young adults [53] and developed messaging specific to youth (https://www.lacedandlethal.com/). Participants in this study shared concerns for young adults who may be less aware of fentanyl-related risks and seek out fentanyl for social reasons. Communication and messaging of harm reduction practices and fentanyl detection resources should be tailored to young people and people who use methamphetamine.

In this study, PWUD recommended increased access to harm reduction and treatment services, including MOUD. Given the increasing saturation of fentanyl in the drug supply in Western states, state public health staff and behavioral healthcare providers should increase the availability of and facilitate access to low-barrier MOUD to reduce harms related to fentanyl [54].

Our study has important strengths and limitations. To our knowledge, this study is the first in Oregon to consider knowledge and response to overdose in the face of fentanyl’s increasing presence in Oregon. Also, our study highlighted the experiences of people most affected by the dangers of fentanyl by explicitly asking PWUD about their preferences for harm reduction and ways to reduce harm. Our study has limitations. First, while our study eligibility criteria included anyone over age 18, in this study, participants under the age of 30 were a minority (*n* = 3). Future research is needed to better understand experiences with fentanyl and suggestions to reduce harm among youth and young adults. Secondly, our sample was limited in its racial diversity. Although white people are the racial majority within Oregon, white participants were overrepresented in our sample relative to the state. Future research is needed to better understand the experiences of people of color, including access to harm reduction and treatment services and experiences with professional first responders. Lastly, fentanyl’s presence in Oregon’s drug supply is rapidly increasing. These data represent a snapshot of the local drug supply, and future research is necessary for continued surveillance of fentanyl and emerging adulterants of threat (e.g., xylazine).

## Conclusion

While illicit fentanyl has subsumed the drug supply in the Northeastern and Midwestern USA, it has more recently reached the Western USA. In this study, PWUD in Oregon reported increased availability of fentanyl pills and heroin, methamphetamine, and other illicit drugs containing fentanyl, and increased fentanyl use by young adults and people who use methamphetamine. PWUD described increased experiences of overdose and continued hesitancy to call 911. These findings demonstrate an urgent need in the Western USA to implement multiple drug checking modalities (e.g., drug checking services and fentanyl test strips), to further investigate the implementation of overdose prevention sites, to improve access to low-barrier substance use disorder treatment and MOUD, and to expand education on fentanyl harm reduction practices and overdose response strategies to PWUD, bystanders, and professional first responders.

## Supplementary Information

**Additional file 1.** **Table S2.** Themes, sub-themes, and supporting quotes.

## Data Availability

The datasets generated and analyzed during the current study are not publicly available for reasons of confidentiality. The qualitative data collected in this study could be used to identify participants and is therefore only available to the research team. It is protected by a Federal Certificate of Confidentiality.

## Abbreviations

EMS: Emergency medical services
HIDTA: High-intensity drug trafficking area
MOUD: Medications for opioid use disorder
OHP: Oregon health plan
PWUD: People who use drugs

## References

[1] O’Donnell J, Tanz LJ, Gladden RM, Davis NL, Bitting J. Trends in and characteristics of drug overdose deaths involving illicitly manufactured Fentanyls—United States, 2019–2020. MMWR Morb Mortal Wkly Rep. 2021;70(50):1740–6. 10.15585/mmwr.mm7050e3 PMC8675656 34914673

[2] Pergolizzi J, Magnusson P, LeQuang JAK, Breve F (2021). Illicitly manufactured fentanyl entering the United States. Cureus.

[3] Oregon-Idaho High Intensity Drug Trafficking Area: Oregon-Idaho HIDTA 2022 Drug Threat Assessment. 2021.

[4] Oregon Health Authority: Opioid overdose in Oregon: Report to the Legislature. Portland, OR; 2021.

[5] Shover CL, Falasinnu TO, Dwyer CL, Santos NB, Cunningham NJ, Freedman RB, Vest NA, Humphreys K (2020). Steep increases in fentanyl-related mortality west of the Mississippi River: recent evidence from county and state surveillance. Drug Alcohol Depend.

[6] Palamar JJ, Ciccarone D, Rutherford C, Keyes KM, Carr TH, Cottler LB (2022). Trends in seizures of powders and pills containing illicit fentanyl in the United States, 2018 through 2021. Drug Alcohol Depend.

[7] Duhart Clarke SE, Kral AH, Zibbell JE (2022). Consuming illicit opioids during a drug overdose epidemic: Illicit fentanyls, drug discernment, and the radical transformation of the illicit opioid market. Int J Drug Policy.

[8] Wagner KD, Harding RW, Kelley R, Labus B, Verdugo SR, Copulsky E, Bowles JM, Mittal ML, Davidson PJ (2019). Post-overdose interventions triggered by calling 911: centering the perspectives of people who use drugs (PWUDs). PLoS ONE.

[9] Bergstein RS, King K, Melendez-Torres GJ, Latimore AD (2021). Refusal to accept emergency medical transport following opioid overdose, and conditions that may promote connections to care. Int J Drug Policy.

[10] Russell C, Ali F, Nafeh F, LeBlanc S, Imtiaz S, Elton-Marshall T, Rehm J (2021). A qualitative examination of substance use service needs among people who use drugs (PWUD) with treatment and service experience in Ontario, Canada. BMC Public Health.

[11] Seaman A, Leichtling G, Stack E, Gray M, Pope J, Larsen JE, Leahy JM, Gelberg L, Korthuis PT (2021). Harm reduction and adaptations among PWUD in rural oregon during COVID-19. AIDS Behav.

[12] Muncan B, Walters SM, Ezell J, Ompad DC: “They look at us like junkies”: influences of drug use stigma on the healthcare engagement of people who inject drugs in New York City. Harm Reduction J. 2020;17:53. 10.1186/s12954-020-00399-8 PMC7393740 32736624

[13] Park JN, Sherman SG, Rouhani S, Morales KB, McKenzie M, Allen ST, Marshall BDL, Green TC (2019). Willingness to use safe consumption spaces among opioid users at high risk of fentanyl overdose in Baltimore, Providence, and Boston. J Urban Health.

[14] Klein KS, Glick SN, Mauro PM (2020). Anticipated use of a supervised drug consumption site among syringe services program clients in King County, Washington: assessing the role of opioid overdose and injection behavior. Drug Alcohol Depend.

[15] Latimore AD, Bergstein RS (2017). "Caught with a body" yet protected by law? Calling 911 for opioid overdose in the context of the Good Samaritan Law. Int J Drug Policy.

[16] Rouhani S, Schneider KE, Rao A, Urquhart GJ, Morris M, LaSalle L, Sherman SG (2021). Perceived vulnerability to overdose-related arrests among people who use drugs in Maryland. Int J Drug Policy.

[17] Substance Abuse and Mental Health Services Administration: key substance use and mental health indicators in the United States: results from the 2020 National Survey on Drug Use and Health. Rockville, MD: Center for Behavioral Health Statistics and Quality; 2021.

[18] Ciccarone D: Fentanyl in the US heroin supply: a rapidly changing risk environment. 2017. 10.1016/j.drugpo.2017.06.010 PMC5742018 28735776

[19] Carroll JJ, Marshall BDL, Rich JD, Green TC (2017). Exposure to fentanyl-contaminated heroin and overdose risk among illicit opioid users in Rhode Island: a mixed methods study. International Journal of Drug Policy.

[20] Mars SG, Bourgois P, Karandinos G, Montero F, Ciccarone D (2016). The Textures of heroin: user perspectives on “black tar” and powder heroin in two U.S. cities. J. Psychoact Drugs.

[21] McLean K, Monnat SM, Rigg K, Sterner GE, Verdery A (2019). “You never know what you’re getting”: opioid users’ perceptions of fentanyl in southwest pennsylvania. Subst Use Misuse.

[22] Cole C, Jones L, McVeigh J, Kicman A, Syed Q, Bellis M (2011). Adulterants in illicit drugs: a review of empirical evidence. Drug Test Anal.

[23] Boyatzis RE (1998). Transforming qualitative information: thematic analysis and code development.

[24] Deterding NM, Waters MC. Flexible coding of in-depth interviews: a twenty-first-century approach. Sociol Methods Res. 2021;50:708–39.

[25] Ciccarone D (2021). The rise of illicit fentanyls, stimulants and the fourth wave of the opioid overdose crisis. Curr Opin Psychiatry.

[26] Karch L, Tobias S, Schmidt C, Doe-Simkins M, Carter N, Salisbury-Afshar E, Carlberg-Racich S (2021). Results from a mobile drug checking pilot program using three technologies in Chicago, IL, USA. Drug Alcohol Depend.

[27] Green TC, Park JN, Gilbert M, McKenzie M, Struth E, Lucas R, Clarke W, Sherman SG (2020). An assessment of the limits of detection, sensitivity and specificity of three devices for public health-based drug checking of fentanyl in street-acquired samples. Int J Drug Policy.

[28] Wallace B, van Roode T, Pagan F, Phillips P, Wagner H, Calder S, Aasen J, Pauly B, Hore D (2020). What is needed for implementing drug checking services in the context of the overdose crisis? A qualitative study to explore perspectives of potential service users. Harm Reduct J.

[29] Karamouzian M, Dohoo C, Forsting S, McNeil R, Kerr T, Lysyshyn M (2018). Evaluation of a fentanyl drug checking service for clients of a supervised injection facility, Vancouver, Canada. Harm Reduct J.

[30] Sutter ME, Gerona RR, Davis MT, Roche BM, Colby DK, Chenoweth JA, Adams AJ, Owen KP, Ford JB, Black HB, Albertson TE (2017). Fatal fentanyl: one pill can kill. Acad Emerg Med.

[31] Latkin CA, Dayton L, Davey-Rothwell MA, Tobin KE (2019). Fentanyl and drug overdose: perceptions of fentanyl risk, overdose risk behaviors, and opportunities for intervention among people who use opioids in Baltimore, USA. Subst Use Misuse.

[32] Suen LW, Davidson PJ, Browne EN, Lambdin BH, Wenger LD, Kral AH (2022). Effect of an unsanctioned safe consumption site in the United States on syringe sharing, rushed injections, and isolated injection drug use: a longitudinal cohort analysis. J Acquir Immune Defic Syndr.

[33] Wood E, Tyndall MW, Montaner JS, Kerr T (2006). Summary of findings from the evaluation of a pilot medically supervised safer injecting facility. Can Med Assoc J.

[34] Kral AH, Lambdin BH, Wenger LD, Davidson PJ (2020). Evaluation of an unsanctioned safe consumption site in the United States. N Engl J Med.

[35] Koester S, Mueller SR, Raville L, Langegger S, Binswanger IA (2017). Why are some people who have received overdose education and naloxone reticent to call Emergency Medical Services in the event of overdose?. Int J Drug Policy.

[36] Wagner KD, Koch B, Bowles JM, Verdugo SR, Harding RW, Davidson PJ (2021). Factors associated with calling 911 for an overdose: an ethnographic decision tree modeling approach. Am J Public Health.

[37] Hamilton L, Davis CS, Kravitz-Wirtz N, Ponicki W, Cerdá M (2021). Good Samaritan laws and overdose mortality in the United States in the fentanyl era. Int J Drug Policy.

[38] Netherland J, Kral AH, Ompad DC, Davis CS, Bluthenthal RN, Dasgupta N, Gilbert M, Morgan R, Wheelock H (2022). Principles and metrics for evaluating oregon’s innovative drug decriminalization measure. J Urban Health.

[39] Davis C, Chang S, Hernandez-Delgado H. Legal interventions to reduce overdose mortality: naloxone access and overdose good Samaritan laws. Edina: The Network for Public Health Law. 2017.

[40] Moss RB, Carlo DJ. Higher doses of naloxone are needed in the synthetic opioid era. Subst Abuse Treat Prevent Policy. 2019;14:6. 10.1186/s13011-019-0195-4 PMC6379922 30777088

[41] Somerville NJ, O'Donnell J, Gladden RM, Zibbell JE, Green TC, Younkin M, Ruiz S, Babakhanlou-Chase H, Chan M, Callis BP (2017). Characteristics of fentanyl overdose—Massachusetts, 2014–2016. MMWR Morb Mortal Wkly Rep.

[42] Hill R, Santhakumar R, Dewey W, Kelly E, Henderson G (2020). Fentanyl depression of respiration: comparison with heroin and morphine. Br J Pharmacol.

[43] Meyerson BE, Russell DM, Kichler M, Atkin T, Fox G, Coles HB (2021). I don't even want to go to the doctor when I get sick now: healthcare experiences and discrimination reported by people who use drugs, Arizona 2019. Int J Drug Policy.

[44] Buajordet I, Naess AC, Jacobsen D, Brørs O (2004). Adverse events after naloxone treatment of episodes of suspected acute opioid overdose. Eur J Emerg Med.

[45] Victor GA, Bailey K, Ray B (2021). Buprenorphine treatment intake and critical encounters following a nonfatal opioid overdose. Subst Use Misuse.

[46] Martin A, Butler K, Chavez T, Herring A, Wakeman S, Hayes BD, Raja A: Beyond buprenorphine: models of follow-up care for opioid use disorder in the emergeny department. West J Emerg Med. 2020;21:257–63. 10.5811/westjem.2020.7.46079 PMC7673896 33207174

[47] Iskander JK, Crosby AE (2021). Implementing the national suicide prevention strategy: time for action to flatten the curve. Prev Med.

[48] Comer SD, Cahill CM (2019). Fentanyl: receptor pharmacology, abuse potential, and implications for treatment. Neurosci Biobehav Rev.

[49] Lipira L, Leichtling G, Cook RR, Leahy JM, Orellana ER, Korthuis PT, Menza TW (2021). Predictors of having naloxone in urban and rural Oregon findings from NHBS and the OR-HOPE study. Drug Alcohol Depend.

[50] Krug A, Hildebrand M, Sun N (2015). "We don't need services. We have no problems": exploring the experiences of young people who inject drugs in accessing harm reduction services. J Int AIDS Soc.

[51] Gunn CM, Maschke A, Harris M, Schoenberger SF, Sampath S, Walley AY, Bagley SM (2021). Age-based preferences for risk communication in the fentanyl era: ‘a lot of people keep seeing other people die and that's not enough for them’. Addiction.

[52] Daniulaityte R, Silverstein SM, Getz K, Juhascik M, McElhinny M, Dudley S (2022). Lay knowledge and practices of methamphetamine use to manage opioid-related overdose risks. Int J Drug Policy.

[53] Banta-Green C, Williams J (2021). Dramatic increases in opioid overdose deaths due to fentanyl among young people in Washington State.

[54] Jakubowski A, Fox A (2020). Defining low-threshold buprenorphine treatment. J Addict Med.

