# Supplementary material for: “It wasn’t here, and now it is. It’s everywhere": fentanyl’s rising presence in Oregon’s drug supply
Source: Harm Reduct J. 2022 Jul 11;19:76. doi: 10.1186/s12954-022-00659-9 (PMC9275036; doi:10.1186/s12954-022-00659-9)
Supplement: Supplementary file 1 — Additional file 1. Table S2. Themes, sub-themes, and supporting quotes. [file 12954_2022_659_MOESM1_ESM.docx]

**Table S2. Themes, sub-themes, and supporting quotes**

| Theme and sub-themes | Quotes (from the text) |
| --- | --- |
| Theme 1: Participant reports of changes in the Oregon drug supply and demographics who use fentanyl | |
| Fentanyl is increasing in the drug supply | *“Three years ago, four years ago, I would have never known to ask if fentanyl was in the heroin I was buying... Today, there’s more fentanyl-heroin than there is just regular heroin. It’s harder to find regular heroin than it is to find fentanyl. Fentanyl has flooded the market.”* |
| Fentanyl pills are widely available | *“It’s [drug supply] been flooded. There’s been a lot of fentanyl, a lot. There are mainly the OxyContin 30-milligram pills, the little, round, blue ones. There’s been a crap-ton of those just flooding the market—a ton of them. Pretty much, if you get an Oxy 30, you know it’s fentanyl. There are no real ones, hardly, going around. It’s just the fentanyl ones.”* |
| Type of people who do drugs are changing | *“a lot of people who were never into opiates, never had a problem with opiates—were just 100 percent meth addicts—they don’t really like it [methamphetamine] now. They don’t hardly ever do meth. It’s all about the fetties [fentanyl pills] and more people—young people, too.”* |
| Theme 2: Experiences with and impressions of professional first responders influenced overdose response behavior | |
| Most participants had experienced, witnessed, or heard of a suspected fentanyl-involved overdose | *“I think everybody who I know who’s a drug user has OD’ed at least once this year.”* |
| Participants were hesitant to call 911 due to fear of arrest | *“Most people who are using have a warrant for their arrest from parole and probation. The last thing anybody’s going to do is call the cops if they don’t have to, so nobody was called.”* |
| Participants shared adaptive overdose response strategies | *“I don’t say “OD [overdose].” I’m saying, “Someone’s having a hard time breathing. Someone’s having complications.” Because if you say “OD,” then they have to notify the police because the police are there to “protect,” quote unquote, the fire department/EMTs.”* |
| Theme 3: Participant recommendations for state and communities | |
| Provide more information about fentanyl | *“It’s really important, I feel, we need to have more information—more insights from doctors’ offices, from meetings, and other places—about fentanyl, and about how it gets mixed with the heroin, it’s really deadly, and all that.”* |
| Increase access and availability of harm reduction services and supplies | *“Little stations where people can go to a safe location to find out if there’s any [fentanyl] in a product. I think that would be cool. Just have private, little spots that have test strips or whatever. People can try them out without the fear of getting in trouble.”* |
| Increase access to substance use disorder treatment services | *“I think that if it [MOUD] could be over-the-counter, it would save so much people. So many people are constantly looking for Subutex, but don’t have Medicare, Medicaid, or OHP [Oregon Health Plan], or a doctor, or something. It could be more readily available or just over-the-counter, even. You would save so much.”* |
| Legalize drug use and regulate the drug supply | *“The only other thing I could possibly think of is complete legalization, and then we’ll actually know what we’re getting.”* |
| Reduce stigma among medical providers | *“…maybe talking to medical professionals, because that’s one of the biggest things. People are afraid to go to the hospital. That’s bullshit. I mean, I’m afraid to go to the hospital because I know how I’m going to get treated.”* |
